# Simultaneous Optimization of Deacetylation Degree and Molar Mass of Chitosan from Shrimp Waste

**DOI:** 10.3390/polym16020170

**Published:** 2024-01-06

**Authors:** Daniel Dumitru Dinculescu, Manuela Rossemary Apetroaei, Cristiana Luminița Gîjiu, Mirela Anton, Laura Enache, Verginica Schröder, Raluca Isopescu, Ileana Rău

**Affiliations:** 1 Faculty of Chemical Engineering and Biotechnologies, National University of Science and Technology POLITEHNICA Bucharest, 011061 Bucharest, Romania; daniel.dinculescu@upb.ro (D.D.D.); anastasiumirela@yahoo.com (M.A.); laura.ena21@gmail.com (L.E.); raluca.isopescu@upb.ro (R.I.); ileana.rau@upb.ro (I.R.); 2 Faculty of Navigation and Naval Management, Mircea cel Bătrân Naval Academy, 900218 Constanta, Romania; manuela.apetroaei@anmb.ro; 3 Faculty of Pharmacy, Ovidius University of Constanta, 900470 Constanta, Romania; virgischroder@yahoo.com

**Keywords:** shrimp waste, chitosan, design of experiments, multiobjective optimization, Pareto front

## Abstract

Shrimp waste is a valuable source for chitin extraction and consequently for chitosan preparation. In the process of obtaining chitosan, a determining step is the chitin deacetylation. The main characteristic of chitosan is the degree of deacetylation, which must be as high as possible. The molar mass is another important parameter that defines its utilizations, and according to these, high or low molar masses are required. The present study is an attempt to optimize the deacetylation step to obtain chitosan with a high degree of deacetylation and high or low molar mass. The study was carried out based on experimental data obtained in the frame of a central composite design where three working parameters were considered: NaOH concentration, liquid:solid ratio, and process duration. The regression models defined for the degree of deacetylation (DD) and for the mean molar mass (MM) of chitosan powders were used in the formulation of optimization problems. The objectives considered were simultaneous maximum DD and maximum/minimum MM for the final chitosan samples. For these purposes, multiobjective optimization problems were formulated and solved using genetic algorithms implemented in Matlab^®^. The multiple optimal solutions represented by trade-offs between the two objectives are presented for each case.

## 1. Introduction

Nowadays, shrimp consumption on the Romanian Black Sea coast has increased considerably due to the population’s awareness of the importance of shrimp meat in a balanced diet. Studies in the literature have shown that regular shrimp consumption can support the immune system, being a rich source of protein and essential nutrients such as vitamins D, B6, B12, and selenium [1,2]. However, a high consumption of shrimps is associated with a large amount of biowaste in the environment, represented by the exoskeletons of these crustaceans, which could become an important source of environmental pollution [3].

The environmental impacts of biowaste resulted from crustacean and mollusk processing in the food industry represent a challenge to seafood exploitation and sustainability. Therefore, the further transformation of waste in usable products becomes a priority. In this respect, the extraction of chitosan from shrimp wastes may be an efficient way to deal with environmental issues and to obtain a useful material with multiple applications. The main characteristics of commercial chitosan are the degree of deacetylation, DD, and mean molar mass, MM, which vary from 70 to 95% and from 10^4^ to 10^6^ Da, respectively. The content of N-deacetylated groups and amino groups in the chitosan structure are also very important in defining chitosan properties. It cannot be neglected that different values for the degree of deacetylation DD as well as for the molar mass MM of the obtained chitosan samples can influence the chitosan performance and its various applications [4,5].

The deacetylation degree of chitosan represents the percentage of *β*-1,4-D-glucosamine units in the biopolymer. These amine groups are obtained by converting the acetamide groups in the glycosidic ring of the polysaccharide by a hydrolysis process using strong alkaline solutions and high temperatures. The chemistry and functionality of chitosan are influenced by two key parameters: the degree of deacetylation (DD) and molar mass (MM), parameters that determine its applications. The degree of deacetylation influences the functional properties of the biopolymer, such as solubility, crystallinity, swelling ratio, bioactivity, and biodegradation. Depending on the degree of deacetylation (DD), chitosan can be classified as either high deacetylation chitosan (HDD) (70–99%) or low deacetylation chitosan (LDD) (55–70%) [6].

Depending on the desired applications, different types of chitosan can be prepared, both oligo and polymeric forms, according to the nature of the raw material and the extraction process [7]. In addition, the functionality of chitosan related to its molar mass is given by its viscosity, solubility, adsorption on solids, breaking strength, and elasticity [8].

At the same time, the molar mass (MM) can be considered a parameter in the classification of chitosan. Thus, it can be found high-molar-mass (HMM) chitosan (MM > 700 kDa), medium-molar-mass (MMM) chitosan (MM between 150 and 700 kDa), low-molar-mass (LMM) chitosan (MM between 50 and 150 kDa), and oligochitosan with MM < 50 kDa [9,10].

In the literature, there are a lot of studies related to the relationship between MM and DD of chitosan and its applications. Thus, the study by Román-Doval et al. [11] showed the connection between different types of chitosan of different molar mass and its antifungal, antiviral, and antibacterial properties with applications in agriculture. The corresponding mechanism of different types of chitosan depends on both MM and DD. Low-molar-mass chitosan, via amino groups, can react much more easily with negatively charged bacterial cell membranes than high-molar-mass chitosan [11]. Joseph et al. [6] reviewed, among other things, the properties and applications of chitosan depending on its molar mass and its degree of deacetylation. They concluded that HDD (high deacetylation degree) chitosan with medium and high molar mass (MMM and HMM) can find applications in areas such as drug delivery systems, scaffolds for tissue engineering, cell immobilization, food packaging, dietary ingredients, etc., while HDD chitosan with low molar mass (LMM) can be used in wastewater treatment, food preservatives, metal reduction, and wound healing. In pharmaceutical or food formulations, high antimicrobial activity types of LDD (low deacetylation degree) chitosan with MMM and HMM can be used, whereas LDD chitosan with LMM and MMM can be used in agriculture, as plant growth promoters, or in applications as inhibitory activity against phytopathogens or for gene/drug delivery [6]. In obtaining chitosan samples with high values of DD (>90%) or, in order to be able to control this characteristic, in the processing of chitin, the concentration of alkaline solutions, the increase in working time, and temperature must be taken into account. The MM values depend on the source of the raw material (shrimps, crabs, fungi, etc.) and can influence the crystallinity, degradation, moisture content, and tensile strength of the biopolymer. The studies of Nuthanid et al. [12] demonstrated that the values for tensile strength and moisture absorption of chitosan samples with similar DD, but with high MM (600–1000 kDa), were higher than those determined for chitosan samples with small MM values (50–60 kDa) [13,14,15,16]. As the degree of deacetylation and the mean molar mass are key characteristics for specific chitosan use, the deacetylation conditions must be controlled and optimized. Optimization techniques generally used in processing biological raw materials are based on adequate experimental programs, and subsequent statistical analysis and modelling in the frame of Response Surface Methodology (RSM) or using neural networks [17]. For instance, Iber et al. [18] used the Box–Behnken experimental program for the optimization of chitosan coagulant from dry legs of giant freshwater prawn, while Younes et al. [19] optimized the chitosan preparation from shrimps starting from experimental data for enzymatic deproteinization obtained in the frame of the Box–Behnken program. In the study of Bajić et al. [20], the link between the composition of the film-forming solution and the properties of the chitosan for food packing design is carried out using RSM modelling and Box–Behnken experimental design. Bello and Olafadehan [21] present an optimization study for central composite design for five factors, using 2^5-1^ factorial points to optimize the properties of chitosan obtained from *Archachatina marginata* shell.

In the present work, the simultaneous optimization of DD and MM for chitosan obtained from shrimp wastes is focused on establishing the optimum operating parameters in the chitin deacetylation process using RSM and multiobjective optimization techniques.

## 2. Materials and Methods

### 2.1. Materials and Investigative Methods

The study was carried out using shrimp wastes, collected from local fishing restaurants, from the Romanian coast of the Black Sea, and frozen before being processed. In order to obtain chitin and, respectively, chitosan by chemical extraction, the first stages of processing these wastes consisted in washing, removing the soft tissues, subsequent drying in the oven (at T = 60 °C, for 3 h) of the obtained exoskeletons, and grinding them until a fine powder was obtained. The reagents used in the extraction procedure were: 4% HCl solutions, obtained from 37% HCl solution, purchased from Chemical Company S.A., Iași, Romania, and 5% NaOH solutions obtained from NaOH pellets, purchased from ChimReactiv SRL, Bucharest, Romania, with purity higher than 99.3%. The acetic acid solution used for chitosan solubilization, ethanol (EtOH) and acetone (p.a.) were purchased from Sigma Aldrich, Taufkirchen, Germany. Commercial chitosan (from shrimp shells) with a molar mass ranging between 190 and 375 kDa, with product number 417963, from Sigma Aldrich was used as a reference.

The DD values of chitosan samples were determined by using potentiometric pH measurements, according to the procedure described in our previous study [22,23,24]. According to Dima et al. [25], the DD values were calculated by using Equations (1) and (2).

(1) $$DD\;(\%)=\frac{203\cdot Q}{1+42\cdot Q}$$

(2) $$Q=\frac{C_{M}\cdot \Delta V}{m}$$

where C_M_ represents the molar concentration of NaOH solution used for titration (mol/L); ΔV is the volume difference between the two inflection points (L); m is the mass of the analyzed chitosan (g); 203 represents the molar mass of chitin (g/mol); and 42 is the molar mass of acetyl group (g/mol).

The viscosimetric method was used for chitosan (M_v_) average molar mass (g/mol) measuring by following a method described in our previous studies by Pădurețu et al. (2019) [22] and Gîjiu et al. (2022) [26]; Mark–Houwink–Sakurada Equation (3) was used for this purpose, based on the intrinsic viscosity value (η) measured (mL/g), where K (13.8 × 10^−3^ mL/g) and α (0.85) are constants that depend on the nature of the solvent, temperature, and chemical structure of the polymer [22,27]:

(3) $$[\eta ]=K\cdot M_{v}^{\alpha }$$

The yield of chitosan was calculated as follows:

(4) $$Yield\;\mathrm{of}\;\mathrm{chitosan}\;(\%)=\frac{Extracted\;\mathrm{chitosan}\;(g)}{Extracted\;\mathrm{chitin}\;(g)}\cdot 100$$

### 2.2. Chitin/Chitosan Extraction Technique Protocol

#### 2.2.1. Chitin Extraction Process

The extraction of chitosan involves four classical steps to obtain it from the raw material (exoskeleton of shrimps). After using three of them (demineralization, deproteinization, and discoloration) the chitin is obtained. The fourth step consists in the deacetylation process and leads to the chitosan obtaining. Chitin extraction from shrimp exoskeletons was carried out following the method described in our previous study [26].

Thus, the demineralization of the shrimp exoskeletons powder was carried out using a 4% HCl solution, added in a ratio of 1:13 (solid: solvent), at room temperature. The mixture was kept for 50 min, under continuous medium stirring. The demineralized powders were obtained by successively washing the product with distilled water until pH ≈ 6.5. The wet samples were dried in the oven (T = 105 ± 2 °C) and weighed.

In the deproteinization step, 5% NaOH solution was added in the ratio of 1:16 (solid/solvent) over the dry demineralized powder. The working parameters of the deproteinization phase were temperature T = 65 ± 1 °C, time = 120 min, and constant medium agitation. The wet chitin powders were successively washed with distilled water until a neutral pH was obtained. Finally, the wet pink chitinous powders were dried. Discoloration of dry chitin powders was carried out with a mixture (1:1 = *v*/*v*) of ethanol and acetone that was added over chitin in a solid-to-solvent ratio of 1:1 (*w*/*v*). Successive washing with distilled water of the wet discolored chitin powder, for 10 min, by removing the alcoholic supernatants and drying the sample in the oven led to obtaining a light yellow-white chitin powder.

Next, the chitin obtained underwent the deacetylation step to obtain chitosan.

#### 2.2.2. Optimization of the Deacetylation Step

Since chitin is known as a semi-crystalline biopolymer, composed of units -(1→4)-2-amine-2-deoxy-d-glucose and -(1→4)-2-acetamine-2-deoxy-d-glucose, the deacetylation process does not take place in a homogeneous and complete step along all chains [28]. Thus, it is conventionally accepted that the percentage of amino groups in the polymer structure represents the boundary between chitin and chitosan. Data from the literature showed that the chitosan obtained through chitin deacetylation (in a strongly alkalized environment and at high temperatures) undergoes a depolymerization process. Motta de Moura et al. [4] mention the variation of the molar mass (MM) from 1100 kDa to 100 kDa, while the deacetylation degree (DD) varied from 67.3 to 95.7%. Both DD and MM depend on the deacetylation condition. As the process is controlled by various operating parameters, the selection of those to be optimized may vary according to specific cases and experimental conditions. The temperature and process duration were considered in [29], and by applying the response surface methodology the minimization of MM was carried out. The optimum conditions identified were a temperature of 130 °C and a duration of 90 min. The chitosan obtained in these conditions was characterized by a deacetylation degree of 90%. Amoo et al. [30] studied the deacetylation of chitin obtained from *Penaeus notialis* shell waste considering the influence of temperature, NaOH solution concentration, and process duration. Based on experimental design and statistical modelling, the optimum conditions for maximum deacetylation yield and maximum DD were identified. For maximum DD, the set of operating parameters identified was: 50% NaOH concentration, 97 °C, and 90 min corresponding to a DD value of 89.7%. Green mussel (*Perna viridis*) shells were used by [31] to obtain chitosan with good yield, high deacetylation degree, and low molar mass. To find out the best operating conditions, several experimental schemes are presented including two different deacetylation conditions: (i) 50% NaOH solution, high temperatures (90–100 °C), 2 h process duration and (ii) 15% NaOH solution, room temperatures, and long durations (24 h). The conclusion was that higher NaOH concentrations favor the increase in DD, while lower concentrations and long duration of the process lead to small values of MM, but also lower DD.

In the present study, the optimization of the deacetylation step of chitin, having as a source the exoskeletons of shrimp wastes, was carried out based on experimental data obtained in the frame of a central composite design. According to Response Surface Methodology, regression models for the degree of deacetylation (DD) and of the mean molar mass (MM) of chitosan powders were built and further used in the formulation of optimization problems. As previously shown, the required chitosan properties depend on its applications, high values are necessary for DD, while high or low values for MM may be advantageous. Consequently, the objectives considered were maximum DD and maximum/minimum MM for the final chitosan samples. The originality of this study consists in the attempt to simultaneously optimize the DD and MM. For these purposes, multiobjective optimization problems were formulated and solved using genetic algorithms implemented in Matlab R2015a.

In the chitin conversion process, the variations of 3 factors were considered: the concentration of the NaOH solution, the ratio between the volume of the NaOH solution and the mass of the chitin powder, as well as the duration of the process. In the present study, the deacetylation temperature was kept the same in all experiments (T = 95 ± 1 °C). The selection of a high temperature value aimed to enable the deacetylation process in shorter durations. In order to maintain the energy consumption at reasonable values, no higher values for the temperature were used.

## 3. Results and Discussion

Following the demineralization and deproteinization steps of the shrimp waste powders, the chitin samples were subjected to deacetylation according to the experimental program mentioned in Table 1, and the obtained chitosan samples were analyzed from the point of view of deacetylation degree, DD, and molar mass, MM, according to the investigative methods presented in Section 2.1.

The degree of deacetylation is a very important structural parameter in the characterization of chitosan because it can influence its solubility, reactivity, and biological properties [32,33]. In addition, the molar mass of the polymer can be influenced by the deacetylation degree value and determines the viscosity of chitosan solutions [34].

### 3.1. Experimental Design

A central composite design, based on a 2^3^ full factorial program was chosen. The range of factors variation is given in Table 1, together with the corresponding values of coded variables.

The experimental measured values for deacetylation degree, DD, and molar mass, MM, for every chitosan sample obtained according to the experimental plan are presented in Table 2, together with the yield obtained in the process.

According to RSM, 2nd degree models, including the pure quadratic terms, were proposed in terms of coded variables for both deacetylation degree and molar mass. The general relation of these models is:

(5) $$y=b_{0}+b_{1}\cdot x_{1}+b_{2}\cdot x_{2}+b_{3}\cdot x_{3}+b_{12}\cdot x_{1}\cdot x_{2}+b_{13}\cdot x_{1}\cdot x_{3}+b_{23}\cdot x_{2}\cdot x_{3}+b_{11}\cdot x_{1}^{2}+b_{22}\cdot x_{2}^{2}+b_{33}\cdot x_{3}^{2}$$

where y stands for deacetylation degree, DD, and molar mass, MM, respectively.

### 3.2. RSM Model for Deacetylation Degree (DD)

The model coefficients were calculated by regression, and the values and their significance are presented in Table 3. According to the Fisher test, the model is significant at a level of 5% (*p* = 0.0052), and the coefficient of determination is R^2^ = 0.91.

**Figure 1:**
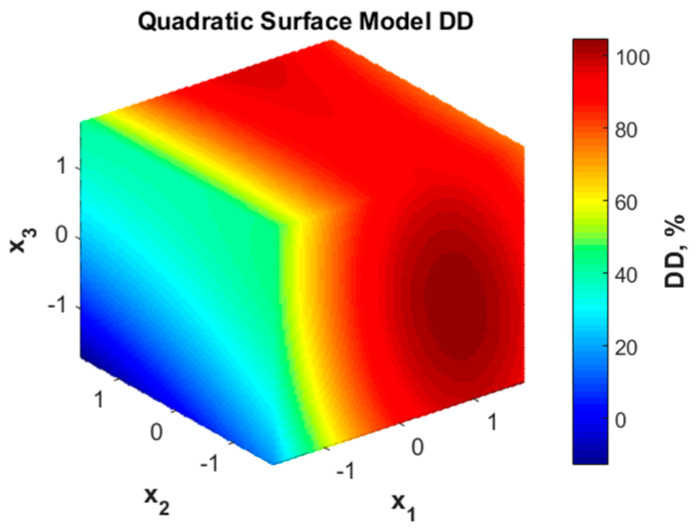
Quadratic surface representation for deacetylation degree (DD) variation with coded variables.

The graphic representation of the variation of the degree of deacetylation with the considered factors is presented in Figure 1. According to this representation, the deacetylation degree value is not favored by low values of x_1_ (NaOH solution concentration) but can reach high values (over 90%) when more concentrated NaOH solutions are used. A less important influence is noticed for variable x_2_ (liquid:solid ratio) as the color code in face x_2_-x_3_ shows. The increase in DD represented by color changes from blue to yellow is noticed only along the x_3_ coordinate, meaning that longer duration (higher x_3_) may favor the deacetylation degree. The minor influence of x_2_ is also confirmed by the analysis of the significance of the model coefficients (Table 3).

As is well known, the elimination of less significant coefficients must be done with much care so as not to drastically decrease the R^2^ value. By eliminating the terms with low significance in the deacetylation degree model, a reduced form is obtained, and this was further used in the optimization step.

(6) $$DD=90.897+16.072\cdot x_{1}-2.066\cdot x_{2}+5.682\cdot x_{3}-3.369\cdot x_{1}\cdot x_{3}-10.823\cdot x_{1}^{2}-3.599\cdot x_{3}^{2}$$

The determination coefficient R^2^ for the reduced model is 0.907, slightly lower than the corresponding value of the full quadratic model.

The linear term in x_2_ showing a slight decrease in the deacetylation degree with x_2_ increase was still maintained in the model, to not completely lose the influence of liquid:solid ratio upon the deacetylation degree.

**Figure 2:**
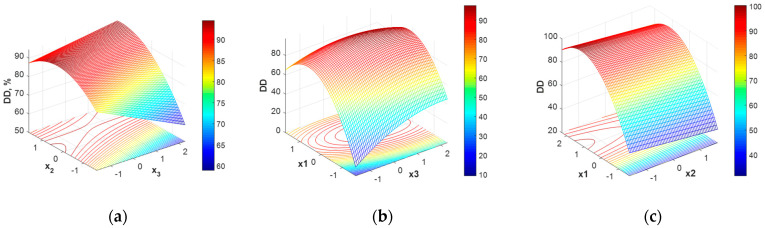
Chitosan deacetylation degree (DD) variation with operating condition expressed in coded values: (**a**) x_1_ = 0, (**b**) x_2_ = 0, (**c**) x_3_ = 0.

The response surface corresponding to this model (Equation (6)) is given in Figure 2.

As Figure 2 shows, the response surface does not exhibit a clear maximum. Some high deacetylation degree values can be reached on a plateau represented by a range of variation for x_1_ and x_3_ (Figure 2b), while x_2_ has a very small influence over the deacetylation degree values (Figure 2c). Analyzing the relative importance of the investigated parameters upon the DD, x_1_ (NaOH solution concentration) proves to mostly influence the DD values, as also mentioned in [35].

### 3.3. RSM Model for Molar Mass (MM)

The general model proposed for molar mass (MM) is given by Equation (5), and the coefficient of the full 2nd-order degree model obtained is given in Table 4. The coefficient of determination is R^2^ = 0.94, and the Fisher test confirms the significance of the model (*p* = 0.0012).

**Figure 3:**
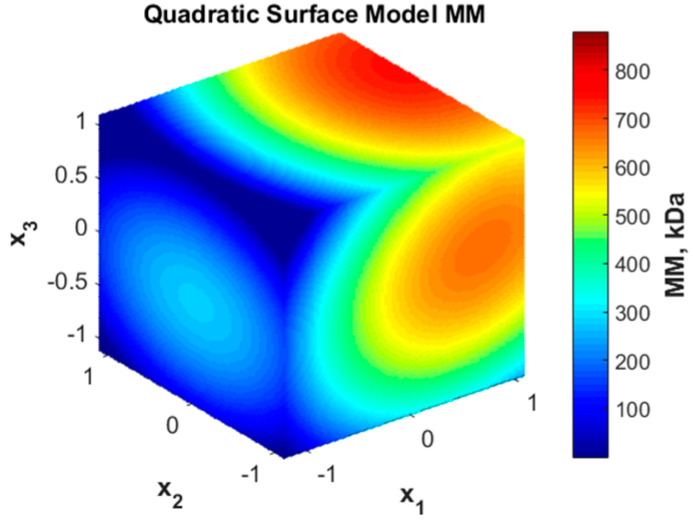
Quadratic surface representation for molar mass (MM) variation with coded variables.

The quadratic representation of the response surface is presented in Figure 3.

As one can see, the molar mass may take both high values, mainly for high concentrations (x_1_), and very low values for long durations (x_3_) and lower concentrations (x_1_).

The model coefficients with very high *p*-values were not considered, and a reduced model was defined with R^2^ = 0.93 and further used in this study.

(7) $$MM=761.559+229.786\cdot x_{1}+41.480\cdot x_{3}+78.312\cdot x_{1}\cdot x_{3}-146.086\cdot x_{1}^{2}-103.142\cdot x_{2}^{2}-202.813\cdot x_{3}^{2}$$

**Figure 4:**
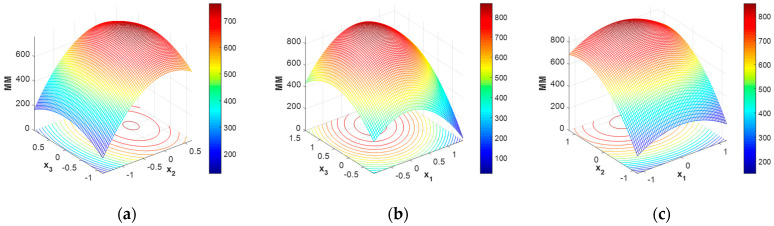
Chitosan molar mass (MM) variation with operating condition expressed in coded values: (**a**) x_1_ = 0, (**b**) x_2_ = 0, (**c**) x_3_ = 0.

A more relevant image of the response surface is given in Figure 4, where the variation of molar mass, MM, according to the reduced model shows a clear maximum.

The maximum value of the molar mass (865 kDa) is identified for the working point defined by coded values of variables: x_1_ = 0.8583, x_2_ = 0, x_3_ = 0.268, which correspond to operating conditions: NaOH concentration = 53.28%, liquid:solid ratio = 18, duration = 128 min.

### 3.4. Solving Multiobjective Optimization for Chitosan Production from Shrimps

Multiobjective optimization is a mathematical approach when the optimal values of decision variables depend on two or more criteria, expressed by corresponding objective functions. Thus, the final decision is taken by applying trade-offs between the conflicting objectives. The final solution resulting in solving such a problem is not unique. There are multiple solutions, of equivalent optimal points, that satisfy the objectives. Any other feasible combination of decision variables would worsen one or the other of the objective functions. The multitude of equivalent optimal solutions build the so-called Pareto front, which may be graphically represented.

In the present study, the two criteria selected refer to the deacetylation degree and the final average molar mass of chitosan. As can be noticed from the analysis of the response surfaces for the two main characteristics of chitosan (Figure 2 and Figure 4), the domains in which the deacetylation degree (DD) is maximum do not overlap, neither with the domain of maximum molar mass (MM) nor with minimum molar mass (MM); therefore, the trade-off solutions will be considered. The formulation of the multiobjective optimization problem was performed in two alternatives:

(a) Maximizing deacetylation degree (DD) and maximizing molar mass (MM)
(b) Maximizing deacetylation degree (DD) and minimizing molar mass (MM)

In both situations, the dichotomy of the objective function is ensured, and applying the genetic algorithm (GA) implemented in Matlab R2015a, a set of equivalent optimal solutions was identified. GAs are a class of metaheuristic techniques based on the generation and selection of decision variable values similar to the natural process of the evolution of a population of individuals. The population size is an important parameter in defining this algorithm. In our study, a population size of 100 is defined. Genetic algorithms are commonly used to generate high-quality solutions to optimization and search.

The objective functions considered are expressed by the polynomial models obtained using experimental design and regression (Equations (6) and (7)).

**Figure 5:**
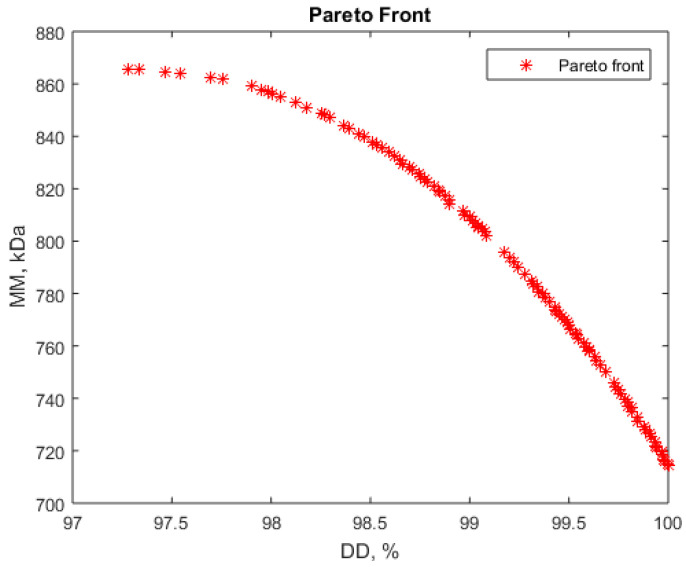
Pareto front for simultaneous maximization of deacetylation degree (DD) and molar mass (MM).

**Figure 6:**
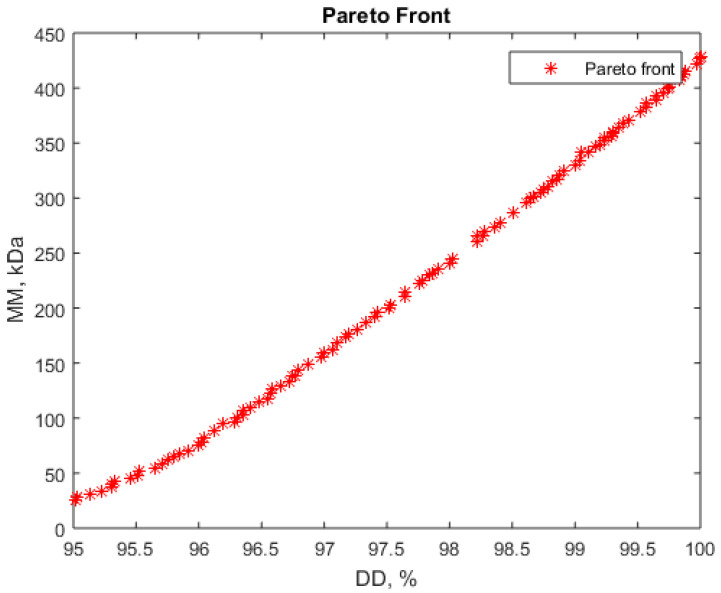
Pareto front for simultaneous maximization of deacetylation degree (DD) and minimization of molar mass (MM).

The results obtained are presented in Figure 5 and Figure 6 where the Pareto fronts for the two problems are given.

Analyzing the Pareto fronts, some possible simultaneous optimization scenarios for deacetylation degree (DD) and molar mass (MM) are possible. For instance, high values for deacetylation degree, between 97.5 and 98.5%, and high molar masses, over 840 kDa, seem possible to achieve (Figure 5). Some values for the vector variables corresponding to the Pareto front are presented in Table 5. As these values show, higher NaOH concentrations (over 50%) and moderate durations, about 120 min, are favorable for such a process.

The liquid:solid ratio (variable x_2_) is less important for the deacetylation degree (DD) control, as previously discussed. This value is about 18, corresponds to the center of the experiment program, and was also identified as the optimum value for maximum molar mass (MM).

The second problem type consists in the maximization of deacetylation degree (DD) and minimization of molar mass (MM).

Even if the RSM model shows that the molar mass has high values over a large range of operating conditions, there are nevertheless some restricted domains where the molar mass is expected to have small values (Figure 4). Thus, the attempt to find some favorable solution to obtain chitosan with a high deacetylation degree but a small molar mass led to some favorable working points.

The Pareto front (Figure 6) shows that low values for molar mass, below 200 kDa, can be obtained only for deacetylation degree below 97.5%, but still in a very good values range. Table 6 presents some sets of operating parameters corresponding to these conditions.

The values from Table 6 (lines 1–4) recommend NaOH concentrations below 50%, very small liquid:solid ratios (close to 9.5, the minimum value considered in our experiments), and long process durations. Some other parameters’ combinations can be identified from the Pareto front. These correspond to very high NaOH concentrations and lower durations (Table 6, line 5).

## 4. Conclusions

The results obtained in the simultaneous maximization of DD and MM are consistent with those reported by [31] that indicate higher NaOH concentration to increase DD, and also with [4], where for the deacetylation of chitin in concentrated NaOH solutions (420 g/L), an increase in process duration is favorable for high DD values. As for MM, the study reported in [29] demonstrates that a high value for MM can be reached at moderate process duration; increased time values would favor the depolymerization and decrease the MM.

For the second problem type, the shorter time for depolymerization required to obtain low MM is compensated by the drastic reaction conditions (high NaOH concentration), but due to the short operating time, the DD may not increase above 95%. A lower duration time and very high temperature were reported by [29] in the attempt to obtain minimum MM, at a constant NaOH concentration of about 41%. They obtained an MM of 150 kDa, while DD was 90%. As the increase in NaOH concentration favors the DD (confirmed also by [36]), the results obtained in the present work may prove that a higher NaOH concentration can compensate for the lower temperature used. In prolonging the deacetylation process, the MM may decrease, while the DD value increases, as also mentioned in [4], so a longer duration may be recommended.

In conclusion, using experimental design, statistical modelling, and multiobjective optimization techniques, we were able to identify the optimum operating conditions in the deacetylation process of chitin obtained from shrimp wastes when two types of materials were envisaged. Thus, for both high DD and MM desired values, NaOH solution concentration around 52%, solution:solid powder ratios of 17–18, and moderate duration (around 2 h) proved to be favorable, while if high DD but low MM are aimed, some recommended operating conditions would be: longer durations (about 3 h), the concentration of NaOH solution slightly below 50%, and a very small liquid:solid ratio.

## Figures and Tables

**Table 1:** Operating conditions investigated for chitin deacetylation to chitosan.

Variables (Operating Parameters)	Coded Variables	Coded Levels and Actual Values				
		−1.68	−1	0	1	1.68
NaOH concentration, %	x_1_	28	35	45	55	62
Liquid:solid ratio	x_2_	9.5	13	18	23	26.5
Duration, min	x_3_	70	90	120	150	170

**Table 2:** Experimental results obtained for chitosan extracted from shrimp waste.

Exp. Nr.	x_1_	x_2_	x_3_	Chitosan Yield, %	DD, %	MM, kDa
1	−1	−1	−1	7.36	62.0	69.83
2	−1	−1	1	9.63	75.5	26.39
3	−1	1	−1	8.41	52.0	66.43
4	−1	1	1	10.09	62.5	67.04
5	1	−1	−1	7.76	100	363.62
6	1	−1	1	8.79	88.5	663.81
7	1	1	−1	7.62	82.95	541.53
8	1	1	1	8.7	91.5	825.00
9	−1.68	0	0	11.57	27.5	34.10
10	1.68	0	0	9.56	92.0	612.14
11	0	−1.68	0	10	88.5	475.43
12	0	1.68	0	9.32	93.77	413.22
13	0	0	−1.68	11.22	63.33	155.52
14	0	0	1.68	11.5	96.95	170.50
15	0	0	0	8.88	96.27	819.99
16	0	0	0	9.43	86.65	804.33
17	0	0	0	10.94	88.25	669.64

**Table 3:** ANOVA results for the deacetylation degree (DD) model.

	Coefficients	Standard Error	*p*-Value
Intercept	90.3432	4.9765	3.81·10^−7^
x_1_	16.0728	2.3384	0.0002
x_2_	−2.0665	2.3384	0.4062
x_3_	5.6821	2.3384	0.0454
x_1_·x_2_	1.1188	3.0539	0.7249
x_1_·x_3_	−3.3688	3.0539	0.3065
x_2_·x_3_	2.1313	3.0539	0.5078
x_1_^2^	−10.6993	2.5761	0.0043
x_2_^2^	0.4207	2.5761	0.8749
x_3_^2^	−3.4749	2.5761	0.2194

**Table 4:** ANOVA results for the molar mass (MM) model.

	Coefficients	Standard Error	*p*-Value
Intercept	761.5591	61.7843	5.31·10^−6^
x_1_	229.7859	29.0314	9.76·10^−5^
x_2_	19.9224	29.0314	0.5146
x_3_	41.4797	29.0314	0.1961
x_1_·x_2_	37.7305	37.9146	0.3528
x_1_·x_3_	78.3116	37.9146	0.0777
x_2_·x_3_	3.4153	37.9146	0.9307
x_1_^2^	−146.0860	31.9832	0.0026
x_2_^2^	−103.1420	31.9832	0.0146
x_3_^2^	−202.8130	31.9832	0.0004

**Table 5:** Parameters’ values selected from the Pareto front in multiobjective optimization considering maximum deacetylation degree (DD) and maximum molar mass (MM).

Variables						Objective Functions	
Coded Values			Real Values			DD, %	MM, kDa
x_1_	x_2_	x_3_	NaOH, %	Liquid/Solid Ratio	Duration, min		
0.80	−0.10	0.31	52.97	17.52	129.33	97.60	863.66
0.86	−0.01	0.25	53.61	17.96	127.54	97.18	865.66
0.75	−0.29	0.34	52.53	16.55	130.18	98.10	853.78
0.78	−0.20	0.29	52.75	17.02	128.76	97.83	860.45

**Table 6:** Parameters’ values selected from the Pareto front in multiobjective optimization considering maximum deacetylation degree (DD) and minimum molar mass (MM).

	Variables						Objective Functions	
No.	Coded Values			Real Values			DD, %	MM, kDa
	x_1_	x_2_	x_3_	NaOH, %	Liquid/Solid Ratio	Duration, min		
1	0.35	−1.67	1.61	48.51	9.64	168.30	96.57	122.4
2	0.36	−1.67	1.43	48.64	9.64	162.80	97.79	225.3
3	0.32	−1.67	1.67	48.16	9.64	170.10	96.00	75.60
4	0.37	−1.68	1.47	48.69	9.62	164.20	97.52	199.7
5	1.07	−1.48	−1.21	55.70	10.55	83.7	94.9	72.5

## Data Availability

Data are contained within the article.
